# Evolution of Complex Niche-Constructing Behaviors and Ecological Inheritance of Adaptive Structures in a Physically Grounded Environment

**DOI:** 10.3389/frobt.2020.00045

**Published:** 2020-04-09

**Authors:** Naoaki Chiba, Reiji Suzuki, Takaya Arita

**Affiliations:** ^1^Graduate School of Information Science, Nagoya University, Nagoya, Japan; ^2^Graduate School of Informatics, Nagoya University, Nagoya, Japan

**Keywords:** niche construction, ecological inheritance, artificial creatures, embodiment, evolution, artificial life

## Abstract

Niche construction is a process in which organisms modify the selection pressures on themselves and others through their ecological activities, and ecological inheritance is the consequence of niche construction inherited through generations. However, it is still unclear how such mutual interactions between robots or embodied agents and their physical environments can yield complex and divergent evolutionary processes or an open-ended evolution. Our purpose is to clarify what kind of complex and various niche-constructing behaviors evolve in a physically grounded environment under various conditions of ecological inheritance of constructed structures and spatial relationships. We focus on a predator-prey relationship, and constructed an evolutionary model in which a prey creature has to avoid predation through the construction of a structure composed of objects in a 2D physically simulated environment supported by a physics engine. We used a deep auto-encoder to extract the defining feature of adaptive structures automatically. The results in the case of no ecological inheritance revealed that the number of available resources can affect the diversity of emerging adaptive structures. Also, in the case with ecological inheritance, it was found that combinations of two types of ecological inheritance, which are the inheritance of adaptive structures and birthplace, can have strong effects on the diversity of emerging structures and the adaptivity of the population. We expect that findings in evolutionary simulations of niche-constructing behavior might contribute to evolutionary design of robotic builders or robot fabrication, especially when we assume physically simulated environments.

## 1. Introduction

All creatures, to a greater or lesser extent, change their own and others' niches through their ecological activities, which modify the selection pressure on themselves and others. This process is called “niche construction” (Odling-Smee et al., 2003; Laland et al., 2016). A typical example of niche-constructing organisms are earthworms that change both the structure and chemistry of soils through their burrowing behaviors. These changes are accumulated over generations, and then bring about different environmental conditions, which expose the successive population to different selection pressure. This effect is also called “ecological inheritance,” as it makes the generation inherit a legacy of modified selection pressures from ancestral organisms (Odling-Smee et al., 2003; Laland et al., 2016).

The effects of niche construction on evolution have been investigated using both mathematical and simple computational models, in which effects of niche-constructing behaviors are represented as changes in variables that represent the quantitative properties of environmental states [e.g., resources (Laland et al., 1996; Han et al., 2009), optimal phenotypic values (Suzuki and Arita, 2010), temperature (Harvey, 2004)]. They clarified various effects of niche construction on evolution in the cases when the environmental state is represented as a quantity (see Chiba et al., 2016).

On the other hand, an important feature of niche construction is that it can create physical and complex structures composed of many components, which cannot be represented quantitatively. Laland et al. (2016) pointed out that exploring a broader range of different possible feedback scenarios might yield new insights into the origin and maintenance of diversity beyond the classic arguments of frequency-dependent selection based on quantitative niche construction. A nest building is a typical and ubiquitous example of such behavior. A beaver makes a dam with branches, which stems the flow of a river and have an influence on many organisms (Odling-Smee et al., 2003). Weber et al. (2013) recently investigated effects of genes on burrowing behaviors of complex tunnels in mice (*Peromyscus*). They showed that the length of the tunnel is affected by at least three genomic regions, while only a single locus affects presence of escape tunnels. This indicates that complex niche-constructing behaviors can have genetic backgrounds and can evolve genetically. While there are a few computational models that focus on such complex and structural properties of niches (Taylor, 2004; Kojima et al., 2014; see Chiba et al., 2016), physically grounded interactions between organisms and environments were not considered.

Our work addresses evolutionary ecology, but also has implications for robotics and evolutionary designs of robot builders. Thanks to recent developments in digital fabrication techniques, the concept of niche construction is likely to become related to evolutionary robotics when considering a combination of evolutionary design of robots and robotic fabrication (Reinhardt and Burry, 2016). Zhang et al. (2018) constructed a 3D printing system that employs multiple mobile robots printing a large, single-piece, structure concurrently. Snooks and Jahn (2018) discussed relationships between multi-agent algorithms and robotic fabrication focusing on feedback between material behavior and robotic operations. We can evolve robots that can solve tasks by creating physical objects online, and such objects can be a scaffold for solving the tasks more easily. This type of behavior of solving problems by modifying external environments is analogous to the concept of niche construction. In other words, we can discuss evolutionary niche-constructing robotics. We expect that findings in evolutionary simulations of niche-constructing behavior might contribute to evolutionary design of robotic builders, especially when we assume physically simulated environments as we will discuss in this paper.

Especially, there are some projects that consider the autonomous evolution of robots using 3D printers such as self-replicating robots (Jones and Straub, 2017) and real-world evolution of robot morphologies (Lipson and Pollack, 2000; Jelisavcic et al., 2017). In such systems, it is possible to consider that the effects of ecological inheritance of constructed materials in ancestral generations can have positive or negative effects on the subsequent generations. Niche construction is recognized as an important factor when considering an open-ended evolution because it can bring about a drive for continued evolution by changing adaptive landscapes (Taylor, 2015). However, it is still unclear how mutual interactions between robots or embodied agents and their complex environments can yield open-ended evolutionary processes via niche construction and ecological inheritance in physical environments.

To clarify the evolutionary dynamics of physically niche-constructing behaviors, we constructed an evolutionary model of virtual organisms in which an organism has to arrive at the goal by performing a physical niche construction that places objects in a 2D physically simulated space with two valleys (Chiba et al., 2016). The results showed that the degree of ecological inheritance, represented as a weathering probability of inherited objects, had a non-linear effect on the adaptivity of the population. That is, the fitness showed a U-shaped curve with the increase in the weathering probability because adaptive structures in a generation tended to become non-adaptive in subsequent generations when constructed structures were unstably inherited. However, the variety in evolved structures was limited because of the simplicity of the task and the analysis of the evolved structures was still preliminary.

Our purpose is to further clarify what kind of complex niche-constructing behaviors can evolve in a physically grounded environment under various conditions of ecological inheritance of constructed structures. There are various types of ecological relationship between species within a shared environment such as predation, competition, mutualism, commensalism, and amensalism. We focus on a predator-prey relationship while ecology in nature would be a combination of them all. This is because the construction of physical structures has been commonly used in both predatory (e.g., creating a trap) and anti-predatory behaviors (e.g., creating a shelter). As a first approach, we particularly focus on the evolution of the latter type of behavior because there is a wide variety of anti-predator adaption including the construction of defensive structures such as beaver dams. We constructed an evolutionary model in which a prey has to avoid predation through the construction of a structure composed of objects in a 2D physical environment, by extending the model in Chiba et al.'s work (Chiba et al., 2016). Nest building or burrowing behaviors are ubiquitous in many taxa (mammals, birds, reptiles, insects, etc.) (Odling-Smee et al., 2003) and nests and burrows have more or less roles of anti-predatory defense for niche-constructing individuals and their offspring. While still abstract, we expect that our experimental setting reflects such situations. We adopted a type of deep auto-encoder (Hinton and Salakhutdinov, 2006) for extracting defining features of emerging adaptive structures.

In the case of no ecological inheritance, we discuss the effects of the number of available resources on the diversity of adaptive structures. In the case with ecological inheritance, we further discuss how the inheritance of spatial relationship between creatures and constructed structures can have a large effect on not only the adaptivity of the population but also on the diversity of emerged adaptive structures.

## 2. Materials and Methods

### 2.1. Evolutionary Model

We constructed a framework for the evolution of physically niche-constructing behaviors based on a predator-prey relationship. In particular, we discuss the evolution of the defensive strategy of a prey based on the construction of physical structures against predation by a predator of which behavior was pre-determined.

#### 2.1.1. Field and Task

We use LiquidFun (Google, 2018), which is an open source physics engine for 2D games, in order to introduce a physically simulated environment into our model. It can simulate physical interactions such as friction and collision between not only rigid-bodies but also a rigid-body and a fluid-object (a soft object to simulate liquid drops or flow), and between fluid-objects.

We assume a x-y coordinate plane that represents the horizontal and vertical space, and there exists gravity along the y-axis toward the bottom. The simulation is updated every time-step *S* (second). Hence, the physical environment is updated 1/*S* times in 1 s. We use the default parameters in the physics engine that define the properties of physical environment with a few modifications^1^.

**Figure 1:**
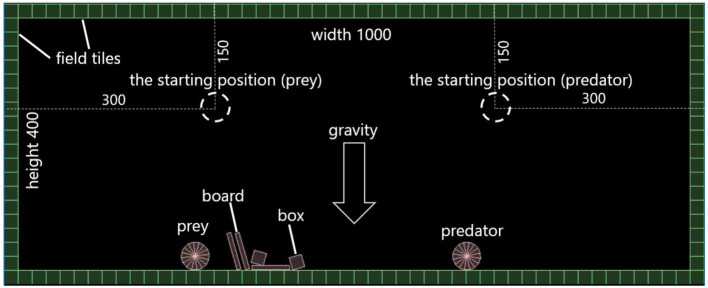
The field for fitness evaluation.

We assumed a 1, 000 × 400 virtual space as shown in Figure 1. A field consists of squared “field tiles” with a side length of 20. A prey and a predator have a circular-shaped body. At first, they are placed on their starting positions and visible from each other (explained later) in the field, which are shown in Figure 1.

The task of the prey is not being captured by the predator. If the prey is captured (touched) by the predator, they are moved back to their starting points. In order to measure the fitness of a prey quantitatively, we assumed that being caught by a predator does not represent complete predation (e.g., being eaten by a predator) but represents incomplete or partial predation having negative effects on the fitness of the prey. Instead, we also assumed that a prey encounters another predator immediately after the occurrence of the incomplete predation event.

Specifically, the fitness is calculated by the following equation (Equation 1):

(1) $$\begin{array}{l} \text{fitness}=\{\begin{array}{ll} 10-\left\{c+\frac{d-d_{f}}{d}\right\} & \left(d_{f}\leq d\right)\\ 10-c & \left(otherwise\right), \end{array} \end{array}$$

where *c* is the number of times for which the prey was captured within the time limit *T* (seconds), *d* is the distance between their starting points, and *d*_*f*_ is the distance between them at the end of fitness evaluation. If the fitness becomes negative, we set it to zero in order to set a lower limit of the fitness to zero. In more detail, 10 is the approximated number of times a predator can catch the prey with the given simulation length and speeds. The first term *c* in the braces in Equation (1) is expected to be equal to or <10. The second term in the braces in Equation (1) reflects the performance of the behavior after the last catch, by measuring the reduced distance between prey and predator. It is normally <1, which is smaller than the decrease in fitness due to being captured. Therefore, the lesser the number of times of being captured is and the larger the distance between a prey and a predator at the end of fitness evaluation is, the higher the fitness of the prey gets.

#### 2.1.2. Prey

**Figure 2:**
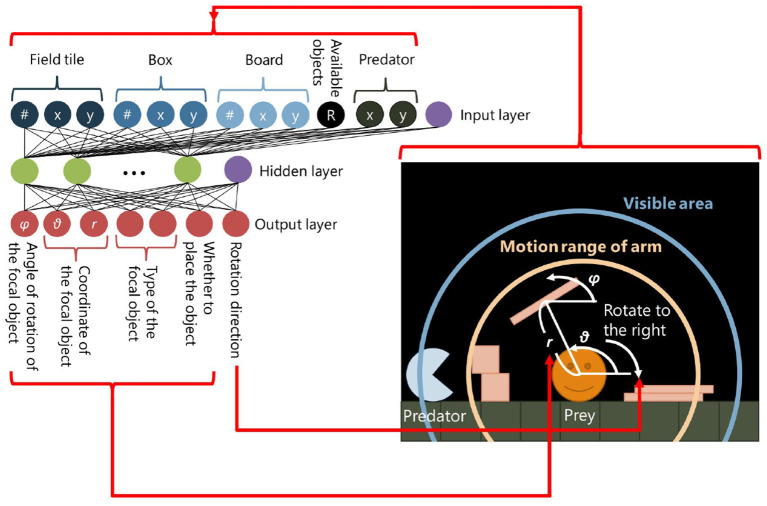
The neural network of a prey.

In our model, a circular-shaped prey with a radius of 20 can move in the field by rotating its body to the left or right. It also can place objects^2^ in the field. This is a niche-constructing behavior in our model in the sense that constructed structures can affect the adaptivity of the prey. There are two types of objects: “box” with a side length of 18 and “board” which is a 6 × 54 rectangle. The prey has two areas around it: the visibility and the motion range of its (invisible) arm (Figure 2, right). The visibility is a round shaped region around the prey with a radius of *F*_*prey*_ and the prey can recognize objects, field tiles, and the predator within this area. The motion range of its arm is also a round-shaped region with a radius of *L*_*prey*_ and the prey can place objects within this area. A placed object will fall on other objects, field tiles, or the predator due to the gravity if it is placed in the air. There is no cost of placing objects.

A three-layer neural network, of which weights are defined by the genotypes of the prey, determines its behavior (Figure 2, left). We use a sigmoid function as an activation function in a hidden and an output layer of the neural network except for neurons which decide a type of an object being placed. The values are input to the neural network every time when the physical environment is updated. The following values are input to the input layer: (1) the number of field tiles, boxes and boards within the visibility; (2) the relative x-y positions of their center of mass from the prey; (3) the relative x-y position of the predator from the prey; and (4) the ratio of the number of remaining objects to its maximum number *B*. The parameter *B* determines the maximum number of the objects that can exist in the field. It reflects the number of available resources for niche-constructing behaviors.

The output layer consists of one neuron which decides the direction of rotation of the prey, which moves by rolling, and the other six neurons are related to placing objects. The first neuron decides the moving direction of the prey. If its output value is higher than 0.5, the torque τ_*prey*_ is applied to the prey in a clockwise direction, otherwise, τ_*prey*_ is applied to it in an anti-clockwise direction. The magnitude of τ_*prey*_ is calculated by the following equation (Equation 2):

(2) $$\begin{array}{l} \text{magnitude of }\tau_{prey}=\frac{100000}{v_{prey}+1}\left(\text{kgf }\cdot \text{ m}\right), \end{array}$$

where *v*_*prey*_ is the current speed of the prey, which is the magnitude of the 2D velocity vector given by the physics engine LiquidFun. This equation represents that the larger the velocity of the prey is, the smaller the applied power becomes, which prevents the velocity of the prey from being too large. We use “*v*_*prey*_ + 1” as a denominator for avoiding division by zero.

The second neuron decides whether the prey places an object or not. If its value is larger than 0.5, which means this neuron is active, the prey places an object in the field, otherwise, it does not, and the output values of the other neurons explained in the following will be ignored.

The third and fourth neurons decide the type of an object which the prey places in the field. If the output value of the third neuron is larger than that of the fourth neuron, the prey places a box, otherwise, it places a board. The fifth and sixth neurons decide the position on which the prey places the object within the motion range of its arm. The position is represented by the polar coordinates as shown in Equation (3):

(3) $$\begin{array}{l} r=L_{prey}\times o_{5}\\ \;\theta =2\pi \times o_{6}, \end{array}$$

where *o*_5_ and *o*_6_ represent the fifth and the sixth output values, respectively. The last neuron decides the angle of rotation of an object being placed, calculated by the following equation (Equation 4):

(4) $$\begin{array}{l} \text{angle of rotation}=2\pi \times o_{7}, \end{array}$$

where *o*_7_ represents the seventh output value. If the focal object will interfere with existing field tiles, objects, predator or prey itself in the field, or will be outside of the field, the action of placing the object is canceled and nothing happens.

#### 2.1.3. Predator

A predator has the same body shape as a prey with the same radius of 20. It can move in the field by rotating its body to the left or right similar to the prey and jump across objects to capture the prey. It also has a round-shaped visible area around it with a radius of *F*_*predator*_ and can recognize the prey within this area.

The behavior of the predator is determined by a fixed algorithm determined a priori as follows. If a prey is not within the visibility of the predator, it does nothing. Otherwise, the predator moves toward the prey. If the prey is on the right side of the predator, the torque τ_*predator*_ is applied to it in a clockwise direction for chasing the prey, otherwise τ_*predator*_ is applied to it in an anti-clockwise direction. The magnitude of τ_*predator*_ is calculated by the following equation (Equation 5):

(5) $$\begin{array}{l} \text{magnitude of }\tau_{predator}=\frac{150000}{v_{predator}+1}\left(\text{kgf }\cdot \text{ m}\right), \end{array}$$

where *v*_*predator*_ is the current speed of the predator, which is the magnitude of the 2D velocity vector given by the physics engine LiquidFun.

If the predator gets stuck in objects and the bottom of it gets in contact with boxes, boards or field tiles, the impulse of which the magnitude is 50, 000(kg · m/s) is applied to it in the elevation angle of 60° toward the prey. This enables the predator to jump across objects.

#### 2.1.4. Evolution

A prey has synaptic weights of its neural network of which values are determined by its own chromosome. Each gene represents a real value of its corresponding weight. The population of prey evolves according to a genetic algorithm.

In the initial generation, there are *N* prey and the values of its genes are randomly assigned between −1.0 and 1.0. After the fitness evaluation of all prey, a pair of parents is selected by a roulette-wheel selection in accordance with the fitness. They produce a pair of two offspring with the same chromosomes as themselves, and a two-point crossover occurs between the chromosomes of offspring with a probability *P*_*c*_. Each gene can mutate with a small probability *P*_*m*_. If a mutation occurs, a random number ∈ [−*R, R*] is added to the value of the gene. This process will be conducted until the number of offspring reaches *N*.

#### 2.1.5. Ecological Inheritance

We introduce an ecological inheritance into the model in order to investigate its effect on the evolution of niche construction. In a pair of offspring, the environmental state of one parent is inherited to the environment of one offspring, and the environmental state of the other parent is also inherited to the other offspring.

Specifically, each offspring inherits the environmental state of the corresponding parent at the end of its fitness evaluation process. There are two different types of ecological inheritance in our model. The first one is an inheritance of the physical structure. All objects in the parent's environment will be copied to the offspring's environment, keeping their types, positions, and rotations the same. However, the degree of inheritance of objects can vary depending on environmental conditions in the real world. Thus, we introduce a probability *W* into our model, which represents a probability of weathering of each object. Each inherited object vanishes according to the probability *W*. Thus, the higher *W* is, the less number of objects the prey inherits.

The second type of ecological inheritance is an inheritance of the spatial relationship in the field from a parent creature. The offspring inherits the place where its parent existed at the end of its fitness evaluation and is born in that place. We call this “inheritance of the birthplace,” hereafter. We introduce a parameter *P* into the model in order to enable or disable inheritance of the birthplace. If the parameter *P* is “True,” the inheritance of the birthplace is enabled and a prey will be born in where its parent was at the end of the fitness evaluation. However, if the parameter *P* is “False,” the inheritance of the birthplace is disabled and all prey will be born in a fixed place.

We conduct the whole process of evolution and ecological inheritance through *G* generations.

### 2.2. Feature Analysis of Adaptive Structures

In order to investigate adaptive structures constructed by the prey in detail, we use a deep auto-encoder (Hinton and Salakhutdinov, 2006) to extract the defining feature of adaptive structures automatically. Such a dimension reduction method has recently been used for analyzing the evolution process of connection weights of embodied agents (Khajehabdollahi and Witkowski, 2018). At first we conducted PCA but it did not work well in that adaptive structures did not distribute widely on the feature space. Thus, we adopted a deep auto-encoder and expected that the reduced dimensions better reflect the global tendency while it might be costly in comparison with some other dimension reduction algorithms (e.g., t-SNE; Maaten and Hinton, 2008).

We conducted multiple experimental trials, each corresponds to the execution of an evolutionary algorithm described above for a fixed number of generations. For every generation in which the best fitness was larger than 9.0 over all the trials, we make a screenshot of the field at the end of the fitness evaluation of the best individual. We re-size these screenshots (1, 000 × 400 pixels) to 125 × 50 pixels and regard them as a data set of the adaptive structures.

**Figure 3:**
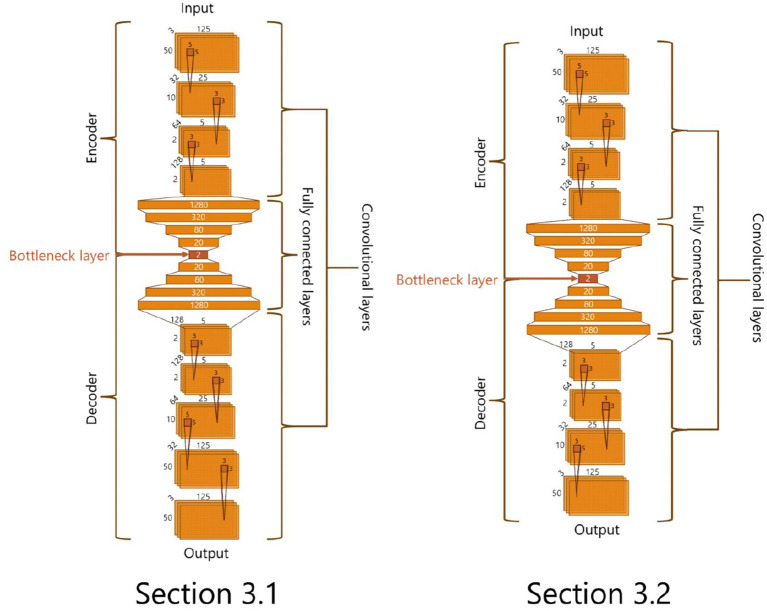
The structures of deep auto encoder.

We conduct an unsupervised feature learning with a deep auto-encoder using Chollet (2015), which is a deep learning library in Python. We use deep neural networks illustrated in Figure 3 for the analyses in sections 3.1 and 3.2. These networks are composed of two parts: an encoder and a decoder part. The former part receives the screenshot of an adaptive structure and reduces its dimension to 2. The output values are then extracted in the decoder part. These networks are trained to reconstruct input screenshots. Through this training, the bottleneck layer of the neural network is expected to represent features of input data (Hinton and Salakhutdinov, 2006). Thus, we use the output of the bottleneck layer composed of two neurons as a feature of the inputted screenshot of an adaptive structure.

**Figure 4:**
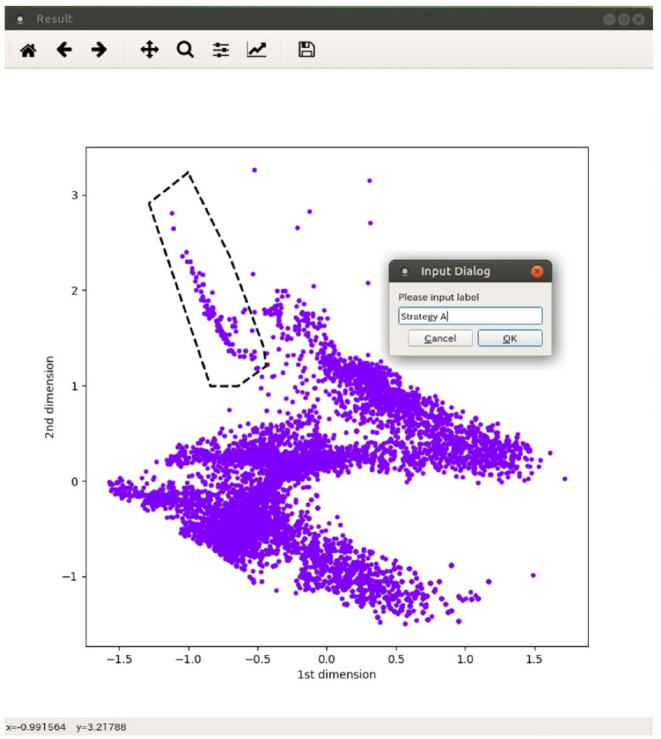
An interface for manual classification of adaptive structures on the feature space.

In the spatial distribution of adaptive structures, there were no clear boundaries between clusters of typically emerging structures, and they were not clearly classified using some clustering algorithms. Thus, we decided to divide the structures into several classes manually by specifying the region of each class on the space, generated by the autoencoder, using an interactive interface for this purpose, as shown in Figure 4. Each structure is represented as a dot in the two-dimensional feature space. The snapshot of each structure pops up when the corresponding dot is clicked by a user to see what types of structures are located on the space. The user can also specify a region with a polygon and determine it as a class with its name. We try to determine the boundary between classes so that the majority of structures exist in the corresponding region of each class. We intend to roughly grasp the distribution of classes of adaptive structures and to estimate their sizes (the number of structures), while this is a manual procedure. Note that the basic idea of this method is initially devised for classification of the spectrograms of bird songs (e.g., an automatic classification of songs with t-SNE and DBSCAN; Sumitani et al., 2018), and is adapted to the classification of adaptive structures in this paper.

## 3. Result

### 3.1. Experiments With No Ecological Inheritance

First, in order to investigate basic behavior of our model and typical defensive structures of prey, we conducted evolutionary experimental trials with no ecological inheritance using the following parameters: *N* = 40; *G* = 3, 000; *S* = 0.02; *T* = 200; *B* = 10, 20, 30, and 40; *F*_*prey*_ = 500; *L*_*prey*_ = 250; *F*_*predator*_ = 1, 000; *P*_*c*_ = 0.7; *P*_*m*_ = 0.001; *R* = 0.003; *W* = 1.0; *P* = *False*.

Because of the extremely high computational cost of conducting multiple experimental trials, we particularly focused on the effects of the parameter *B*, which is the number of available objects for niche-constructing behavior, on evolution, as one of the important factors that reflects the richness of environments for niche-constructing organisms. Thus, we conducted 10 trials for each parameter setting of *B*.

#### 3.1.1. Basic Analysis

**Figure 5:**
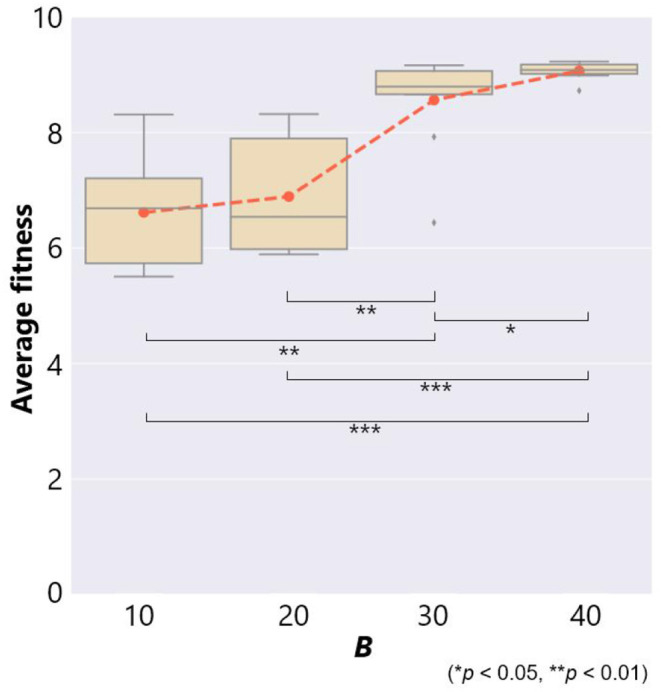
The fitness and the maximum number of objects *B* in the case of no ecological inheritance. Symbols denote statistical comparisons of the fitness between cases of *B* using the Wilcoxon rank sum test.

Figure 5 shows the average fitness over all the trials for each case of the maximum number of objects *B*. The horizontal axis represents *B* and red dots represent the average fitness. We used the fitness values of the last 1, 500 generations for calculating the average fitness in each trial to eliminate the effects of initial conditions.

This figure also shows a box plot of the fitness in each case of 10 trials. There were significant differences in the fitness except for the case between *B* = 10 and 20. When a prey was able to use a smaller number of objects, *B* = 10 and 20, the average fitness was 6.61 and 6.89, respectively. This means that the prey was captured by a predator several times. On the other hand, when the prey was able to use more objects, *B* = 30 and 40, the average fitness was 8.56 and 9.07, respectively. These results show that prey evolved not to be captured by a predator if they could use a larger number of objects.

**Figure 6:**
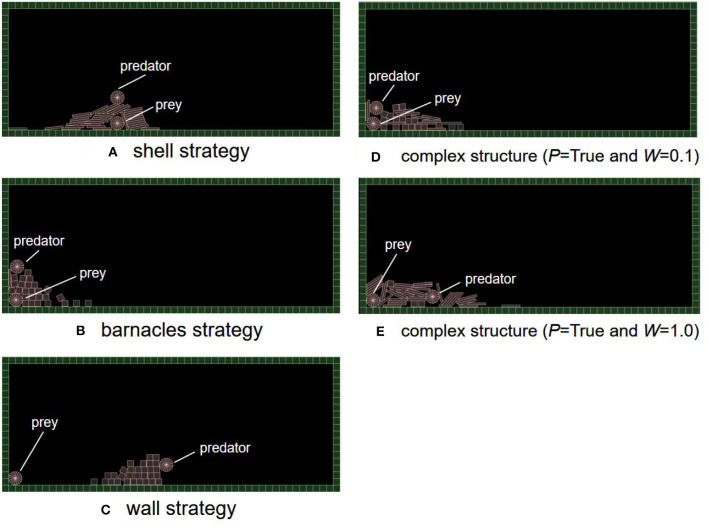
The typical strategies which evolved in our model. **(A)** Shell strategy, **(B)** barnacles strategy, **(C)** wall strategy, **(D)** complex structure (*P* = True and *W* = 0.1), and **(E)** complex structure (*P* = True and *W* = 1.0).

It turned out that three typical and adaptive strategies emerged in the experimental trials. Figure 6 shows their snapshots during their fitness evaluations. See Supplemental Videos (V1: shell, V2: barnacles, V3: wall, and V4: complex). A “shell strategy,” like a shellfish, encloses the whole body of a prey with many objects without moving (Figure 6A). A “barnacles strategy” uses both field tiles and objects to enclose the whole body of a prey by moving toward the left (Figure 6B). A “wall strategy” creates a wall between a prey and a predator (Figure 6C). These strategies could be recognized as “primary or secondary defense” in a predator-prey relationship (Edmunds, 1974) in the sense that the prey was prevented from being captured by a predator using the adaptive structure. Thus, we mainly focus on how the experimental conditions can affect the emergence of these adaptive structures using the feature analysis.

In addition, Figures 6D,E also show examples of complex structures in the cases of the inheritance of birthplace and/or objects. In the case of the inheritance of birthplace and boards (Figure 6D, *P* = *True* and *W* = 0.1), the structure was composed of both boxes and boards. This prey placed multiple box and boards when a predator climbed the inherited structures and was going to touch the prey. Also, in the case of the inheritance of birthplace only (Figure 6E, *P* = *True* and *W* = 1.0), the structure looked like the one of a barnacle strategy but the predator was locked in a pile of boards. In these cases, a small difference in the angle or positions of objects being placed could affect whether the prey survives or not.

#### 3.1.2. The Feature Analysis of Adaptive Structures

**Figure 7:**
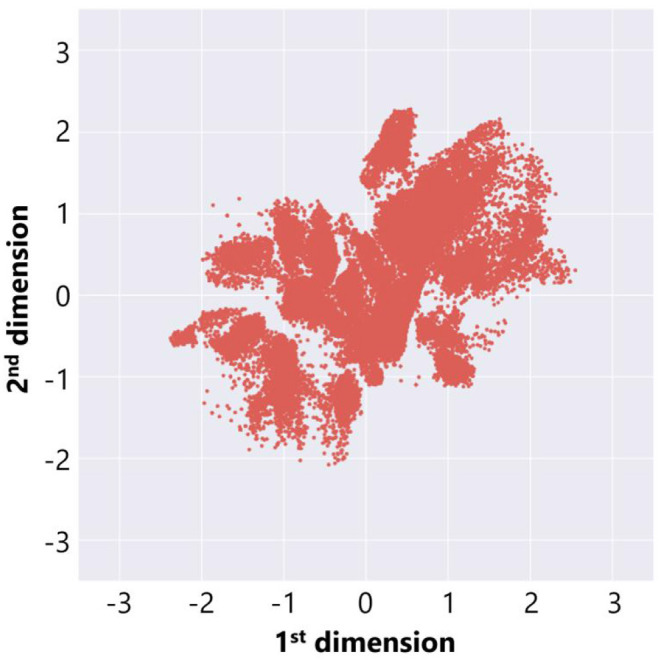
The feature plot of adaptive structures in the case without ecological inheritance.

In order to extract features from the adaptive structures, we made a data set of 72, 062 adaptive structures from all trials above and trained a deep neural network in Figure 3 (left) in the procedure described before. We use the parameters and settings of learning processes with Keras as follows: activation function: hyperbolic tangent; optimization function: adadelta; loss function: binary cross entropy; batch size: 10; and epochs: 200 for section 3.1 and 2,000 for section 3.2. We found that the error rate (loss) did not decrease so significantly and it was expected to be due to the large size and variations in our data set. Thus, we decided to adopt a result from multiple learning trials that showed a relatively clear distribution of adaptive structures. Figure 7 represents the distribution of the adaptive structures in a two-dimensional feature space and we can see that there were some clusters in the feature space.

**Figure 8:**
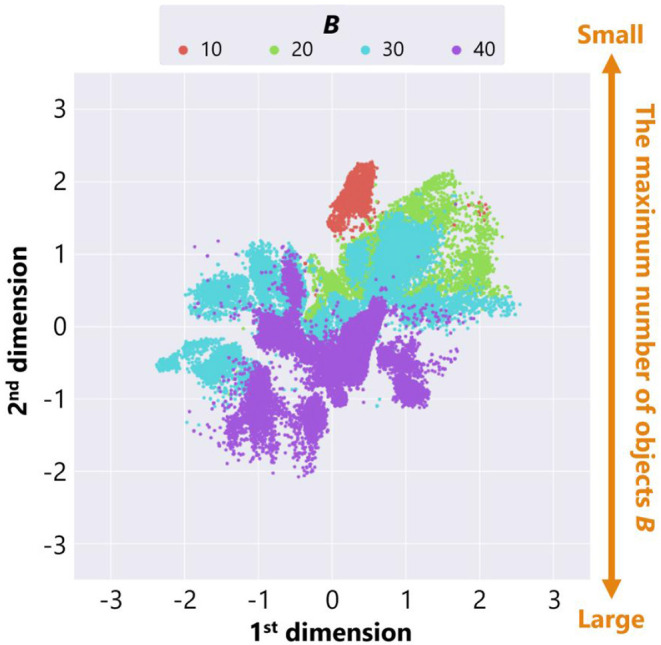
The feature plot of adaptive structures colored by the parameter setting of the maximum number of objects *B* in the case of no ecological inheritance.

**Figure 9:**
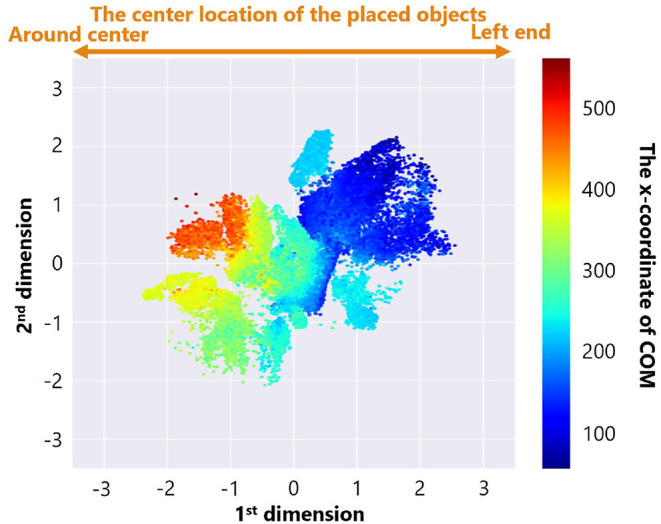
The feature plot of adaptive structures colored by the x-coordinate of COM of each adaptive structure in the case of no ecological inheritance.

Figure 8 is a feature plot of adaptive structures colored by the parameter setting of the maximum number of objects *B*. It can be seen that *B* became larger as the y-coordinate value of each point decreased. This means that a vertical axis approximately reflects the parameter setting of *B*. On the other hand, Figure 9 is a feature plot of adaptive structures colored by the x-coordinate of the center of mass (COM) of each adaptive structure (i.e., the COM of the all placed objects). We can see that the color of each point changed from blue (i.e., the x-coordinate of COM was small) to red (i.e., the x-coordinate of COM was large) as the x-coordinate of the point decreased. This indicates that the horizontal axis approximately reflects the center of mass of the placed objects.

These analyses showed that our trained neural network extracted two features, the parameter setting of the maximum number of objects *B* and the x-coordinate of COM of the placed objects. That is, the experimental condition can strongly affect emerging adaptive structures.

**Figure 10:**
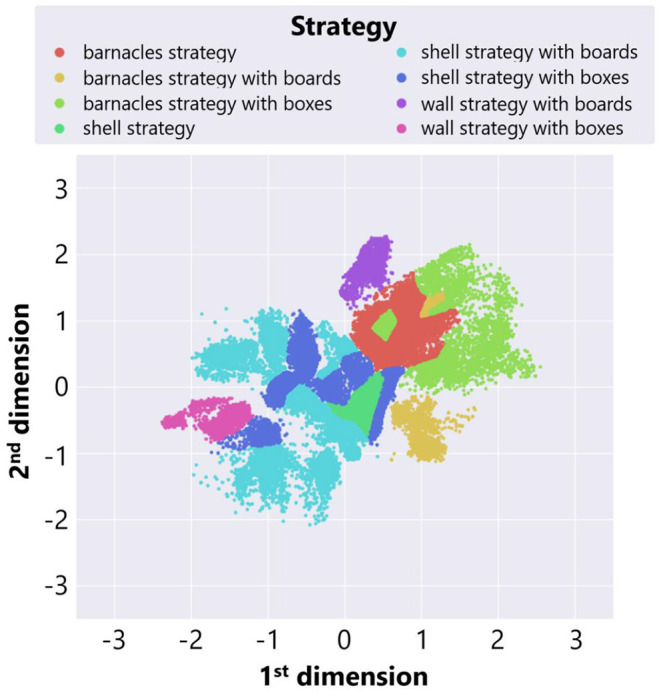
The result of manual classification of adaptive structures in the case of no ecological inheritance.

We also roughly classified adaptive structures into three typical strategies explained before focusing on types of objects used in a constructed structure. Figure 10 shows the result of manual classification. We investigated each region in a feature space and labeled the points in each region as a strategy which are expected to be the most commonly observed in the region. Each color represents the type of adaptive structure. We regarded the area in which there were changes in the dominant structures and the composition of blocks as boundaries. While the interactive interface for classification (Figure 4) helped us with visually inspecting snapshots of adaptive structures around the boundaries, there could be some subjective bias in the classification results around boundaries. We expect that more sophisticated algorithms for embedding might contribute to this potential problem.

We see that barnacles strategies were located to the left side of the feature space, while shell strategies were located to the right side. This result was consistent with the fact that the center location of a shell strategy was close to the center of the field, whereas that of the barnacles strategy was to the left end of the field. Also, there were several types in the same strategy focusing on the types of placed objects. For example, both a “shell strategy with boxes” and a “shell strategy with boards” are shell strategy, but components of adaptive structures are different between these strategies.

Moreover, we can see that locations of a wall strategy varied depending on the types of placed objects. This is because the center locations of adaptive structures were different between these wall strategies. A “wall strategy with boxes” created a tall wall around the center, whereas a “wall strategy with boards” created a low wall near the left end. Thus, the characteristics of these two strategies were reflected in the location of each strategy in the feature space.

**Figure 11:**
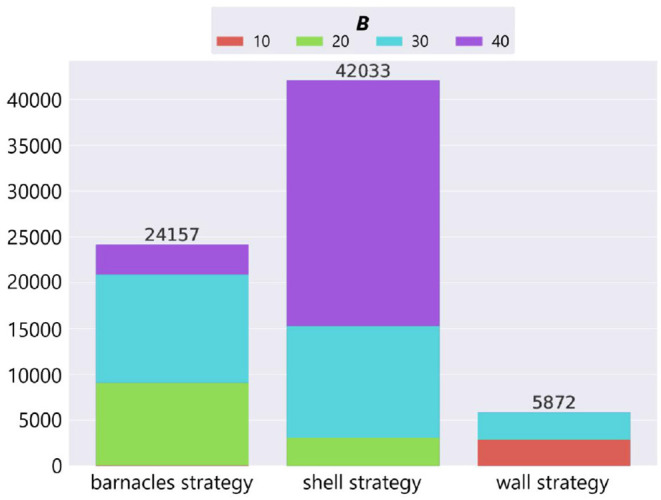
The number of adaptive structures of each strategy in the case of no ecological inheritance.

We counted the number of adaptive structures of each strategy based on the result of manual classification (Figure 11). The horizontal axis represents strategies, the vertical axis represents the number of adaptive structures, and the color represents the parameter setting of the maximum number of objects *B*.

In the cases where the number of resources was small (*B* = 20, 30), the barnacles strategy, which utilizes components of the environment (i.e., field tiles), was likely to evolve. In the case where the number of resources was large (*B* = 40), the shell strategy, which uses a lot of resources without moving, was likely to evolve. On the other hand, the wall strategy was likely to evolve in specific conditions (i.e., *B* = 10 and 30). It should be noted that when the number of available resources was intermediate (*B* = 30), all the strategies in our model emerged.

In sum, the environmental condition can significantly affect the diversity of emerging adaptive structures in terms of large variations in emerging typical structures. The intermediate degree of the richness of environments could bring about the highest diversity of structures. Also, a feature extraction based on unsupervised feature learning can contribute to a broad understanding of the distribution and estimation of the dominant structures.

### 3.2. Experiments With Ecological Inheritance

Next, in order to examine how ecological inheritance affects the evolution of adaptive structures, we conducted evolutionary experiments with ecological inheritance of constructed niches focusing on the parameters *W* and *P*. We used the following parameters: *N* = 40; *G* = 1, 000; *S* = 0.02; *T* = 200; *B* = 10, 20, 30, and 40; *F*_*prey*_ = 500; *L*_*prey*_ = 250; *F*_*predator*_ = 1, 000; *P*_*c*_ = 0.7; *P*_*m*_ = 0.001; *R* = 0.003; *W* = 0.01, 0.1, and 1.0; *P* = *False* and *True*. We used *G* = 1, 000 instead of *G* = 3, 000 for reducing the large computational cost of conducting multiple trials with various combinations of settings, but we observed that the population almost converged by the end of each trial. We conducted 5 trials for each combination of the parameters *B, W*, and *P*.

**Figure 12:**
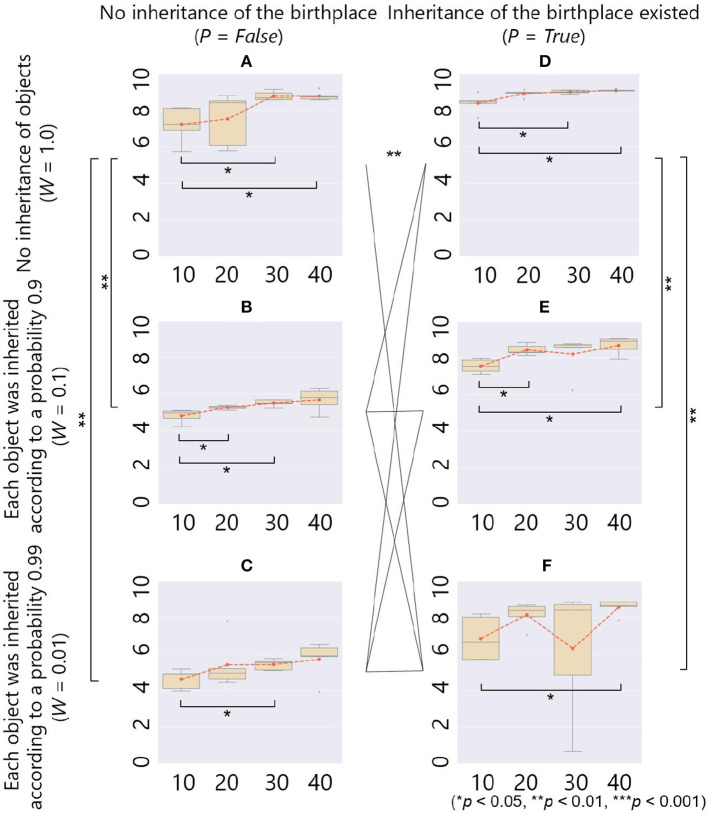
The fitness (y-axis) and the maximum number of objects *B* (x-axis) in the case of ecological inheritance. Symbols denote statistical comparisons of the fitness between possible cases of ecological inheritance (*W* and *P*) using the Wilcoxon rank sum test. We aggregated values of the fitness among all conditions of *B* for each case of ecological inheritance, and conducted the test on the aggregated fitness values. **(A)** *W* = 1.0, *P* = False; **(B)** *W* = 0.1, *P* = False; **(C)** *W* = 0.01, *P* = False; **(D)** *W* = 1.0, *P* = True; **(E)** *W* = 0.1, *P* = True; and **(F)** *W* = 0.01, *P* = True.

Each box plot in Figure 12 shows a relationship between average fitness on the whole population in the last 500 generations (vertical axis) and parameter setting of *B* (horizontal axis) in each combination of the parameters *W* and *P*. In each box plot, there was a significant difference in the fitness between the case with *B* = 10 and some other cases of *B* (= 20, 30, and 40), showing that the smaller number of objects had significantly negative effects on the fitness.

As for the comparison between box plots, in the case of *P* = *False* and *W* = 1.0, in which there was no ecological inheritance and the setting of parameters was the same as that of the previous section except for the number of generation *G*, Figure 12A shows a positive relationship between *B* and the average fitness as with the cases of no ecological inheritance (Figure 5). In the cases of *P* = *False*, and *W* = 0.1 or 0.01, in which when placed objects were inherited, each of them vanished according to a probability 0.1 or 0.01, respectively, there was also a positive relationship between *B* and average fitness (Figures 12B,C). However, when compared to the cases without ecological inheritance, the average fitness was very low in both cases of weathering probability. This result shows a negative effect of the inheritance of objects on the adaptivity of the prey.

On the other hand, in the case of *P* = *True* and *W* = 1.0, in which there was only an inheritance of the birthplace, the average fitness was the highest in all cases of the parameter settings. This is expected to be that the inherited place was a beneficial environment for constructing an adaptive structure from scratch. In the cases of *P* = *True*, and *W* = 0.1 or 0.01, in which there was the inheritance of both objects and birthplace, Figures 12E,F show that the average fitness was higher than the case with inheritance of objects only (Figures 12B,C). These results show that the inheritance of the birthplace combined with the inheritance of objects had a positive effect on the adaptivity of the prey. Even in these cases, the evolved niche-constructing behavior was important to repair the inherited structures and keep them adaptive because they can be broken by an occasional weathering of some objects and an attack by a predator. The statistical analyses showed that there were significant differences in the fitness between the cases with (*W* = 0.1 and 0.01) and without (*W* = 1.0) ecological inheritance of objects, and the ecological inheritance of the birthplace also had significant effects on the fitness, as mentioned above.

The reason why the inheritance of objects had a stronger negative effect on the adaptivity of the prey in the cases without inheritance of the birthplace was that the prey was born outside of a structure constructed by its ancestors. In this case, the prey is not able to utilize the structure created by the shell and the barnacles strategies because it is hard to enter into them. Therefore, the prey was captured several times by a predator and thus got very low fitness. On the other hand, the inheritance of the birthplace enabled prey to born inside of the constructed structure because its ancestors were also born inside of it and thus it was able to use the adaptive structure. That is why the inheritance of the birthplace had a positive effect on the adaptivity of the prey in our model. In other words, the inheritance of the birthplace maintained adaptivity of inherited structures.

**Figure 13:**
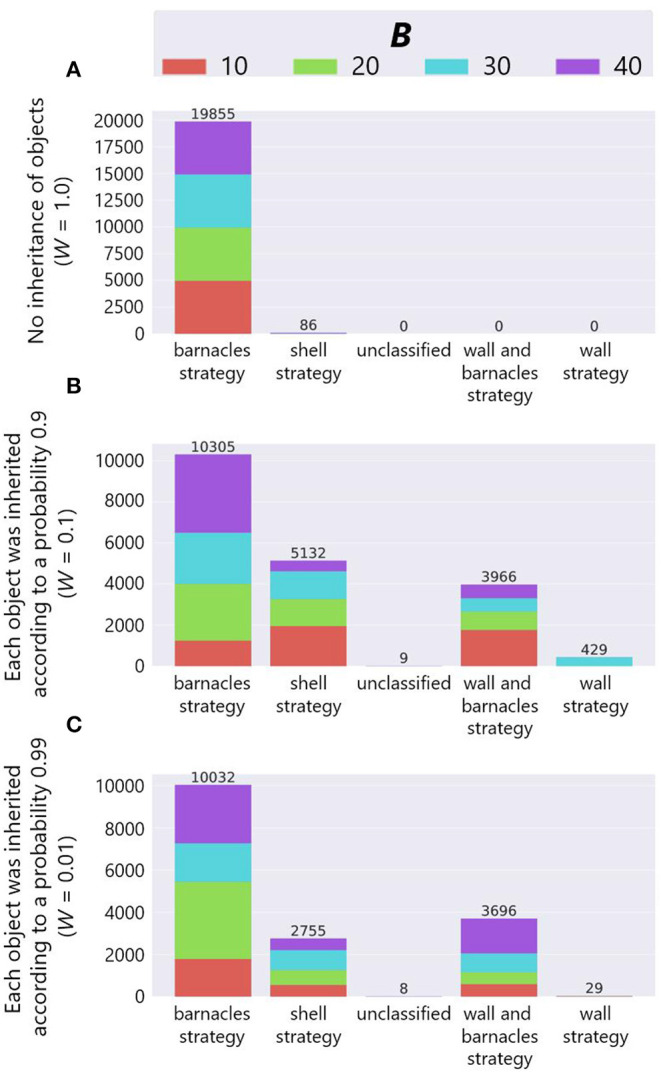
The results of manual classification in the case of inheritance of the birthplace. **(A)** *W* = 1.0, **(B)** *W* = 0.1 and **(C)** *W* = 0.01.

The combined effect of the inheritance of objects and birthplace also brought about the diversity of adaptive structures. Figure 13 shows the number of adaptive structures of each strategy in each combination of the parameter *W* and *B* when *P* = *True*^3^. In the bar charts, the horizontal axis represents strategies, the vertical axis represents the number of adaptive structures, and color represents the parameter setting of the maximum number of objects *B* as in Figure 11. Note that we combined some wall and barnacles strategies in one category because that category could not be divided into two dominant categories of two strategies by manual inspection, and also added “unclassified” category in which their structures could not be classified into any of the typical strategies.

We see that while the most of adaptive structures were barnacles strategies in the case of no ecological inheritance of objects (*W* = 1.0), there were more shell strategies when there exists the ecological inheritance of objects (*W* = 0.1 and 0.01). This means that the succeeded structures and the spatial relationship from the parent organisms were expected to affect the construction of structures, and further affected the evolutionary dynamics of niche-constructing behavior, making it more diverse.

In addition, we also see that barnacles strategy was still dominant when *W* = 0.1 and 0.01. This is might be due to the robustness of the barnacles strategy against external perturbations such as stochastic weathering of objects or physical effects from a predator, because the barnacles strategy makes use of field tiles that cannot be collapsed by such external factors.

## 4. Conclusion

In order to investigate the evolution of complex niche-constructing behaviors in a physically grounded environment, we developed a framework of an evolutionary model in which a virtual organism can construct structures by placing objects in a 2D physically simulated environment. We assumed a task in which a prey has to avoid predation through the construction of structures composed of objects, and evolved the strategy of prey organisms with and without ecological inheritance of objects and birthplace.

Evolutionary experiments without ecological inheritance showed that three typical strategies evolved depending on the maximum number of objects, affecting the adaptivity of the population. The proposed feature extraction techniques contributed to the classification of evolved adaptive structures. We also showed that there was a large diversity in the evolved adaptive structures when the number of objects was intermediate. These results indicate that the number of environmental resources can affect the diversity of emerging adaptive structures.

In the case with ecological inheritance, the inheritance of the birthplace increased the average fitness whereas the inheritance of objects decreased it. In addition, the combination of the inheritance of environmental structures and spatial relationships brought about diversity in emerging adaptive structures, showing that conditions of ecological inheritance can have strong effects on the divergent evolutionary dynamics of complex niche-constructing behaviors. This implies that both complex niche construction and ecological inheritance can significantly contribute to an open-ended evolutionary process in real and physically grounded environments, such as long-term embodied evolution of robots.

Future work includes conducting feature analyses using different dimensionality reduction algorithms to see if adaptive structures can be more clearly clustered, experiments with multiple tasks (e.g., anti-predatory and foraging behavior) and different abilities of prey (e.g., removal of placed objects) and coevolution of prey and predator species. Another future directions is to consider an application of this framework to more ecologically grounded questions, which might not be clearly discussed from empirical data. For example, it is reported that hermit crabs can only survive in remodeled shells handed down from conspecifics, implying that inherited niche (remodeled shells) can affect evolution of social relationships (Laidre, 2012). We would be able to discuss such complex interplays between biological and cultural evolution processes by extending our framework to multi-agent systems.

## Data Availability Statement

The datasets generated for this study are available on request to the corresponding author.

## Author Contributions

NC, RS, and TA designed the experimental procedures, conducted experiments, analyzed the results, and wrote the manuscript.

### Conflict of Interest

The authors declare that the research was conducted in the absence of any commercial or financial relationships that could be construed as a potential conflict of interest.

## Footnotes

^1^Gravity *g* = 9.8(m/s^2^), density ρ = 1.0(kg/m^2^), coefficient of friction of a prey and a predator μ_*i*_ = 0.7 and coefficient of friction μ = 0.3.

^2^In this paper, the term “objects” represents boxes and boards placed by the prey. It does not include field tiles.

^3^We conducted classification processes of adaptive structures on the set of evolved adaptive structures when *W* = 1.0 and *W* = (0.1 and 0.01), in similar manner to the one in the previous section.

## Supplementary Material

Supplemental Video 1 Shell strategy.

Supplemental Video 2 Barnacles strategy.

Supplemental Video 3 Wall strategy.

Supplemental Video 4 Complex structure (*P* = True and *W* = 0.1).

Supplemental Video 5 Complex structure (*P* = True and *W* = 1.0).
